# Intraventricular iron causes severe hydrocephalus – a model of severe neonatal hydrocephalus

**DOI:** 10.1186/s12987-025-00745-7

**Published:** 2025-12-24

**Authors:** Kwang-Min Kim, Arokoruba Oboba Cheetham-West, Mohamed Rafiuddin Ahmed, Megan Phillips, Andrey V. Malkovskiy, Venkata Raveendra Pothineni, Kyle D. Brewer, Chirag B. Patel, Jayakumar Rajadas, Kelly B. Mahaney

**Affiliations:** 1 Department of Neurosurgery, Stanford University School of Medicine, Stanford, CA 94305 USA; 2 Department of Physiology, Gachon University College of Medicine, Incheon, 21999 Korea; 3 Department of Pharmacology, Vanderbilt University, Nashville, TN 37232 USA; 4 Department of Plant Biology, Carnegie Institute of Washington, Stanford, CA 94305 USA; 5 Department of Medicine, Stanford University School of Medicine, Stanford, CA 94305 USA; 6 Department of Neuro-Oncology, The University of Texas MD Anderson Cancer Center, Houston, TX 77030 USA; 7 Cancer Biology Program, The University of Texas MD Anderson Cancer Center, The University of Texas Health Science Center at Houston Graduate School of Biomedical Sciences, Houston, TX 77030 USA; 8 Neuroscience Graduate Program, The University of Texas MD Anderson Cancer Center, The University of Texas Health Science Center at Houston Graduate School of Biomedical Sciences, Houston, TX 77030 USA; 9 Advanced Drug Delivery and Regenerative Biomaterials Laboratory, Cardiovascular Institute, Stanford University School of Medicine, Stanford, CA 94305 USA

**Keywords:** Intraventricular hemorrhage, Post-hemorrhagic hydrocephalus, Germinal matrix hemorrhage, Iron, Choroid plexus, Ependyma, Pre-clinical model

## Abstract

**Background:**

Neonatal germinal matrix hemorrhage-intraventricular hemorrhage and subsequent post-hemorrhagic hydrocephalus (PHH) is a leading cause of neurologic morbidity and mortality in preterm infants. Preclinical models of PHH have implicated hemoglobin and iron-mediated cellular injury in the pathogenesis of PHH, but have typically demonstrated a phenotype characterized by mild to moderate ventriculomegaly – the equivalent of which may not even require intervention in the human condition. We sought to establish a preclinical model of severe IVH that recapitulates the severity of the hydrocephalus phenotype requiring neurosurgical intervention and to determine the causal blood degradation product resulting in severe hydrocephalus following preterm IVH.

**Methods:**

Sterile saline 7.5 µL/g (*n* = 20), hemoglobin (Hb) 120 mg/mL (*n* = 25), iron 1.2 mg/mL (FeCl_3_) (*n* = 49), or lysed red blood cells (RBC) 7.5 µL/g (*n* = 41) were injected bilaterally into the lateral ventricles of postnatal day 3 Sprague Dawley rat pups. Ventricular volumes were assessed at postnatal days 5, 12, 19 and 26 with 11.7 T MRI and compared between groups. Ventricular ependyma and choroid plexus morphology were characterized with SEM. Ependymal and choroid plexus iron deposition were evaluated with Perls stain. Clinical and behavioral manifestations of hydrocephalus were monitored.

**Results:**

Lysed RBC and iron intraventricular injections resulted in severe hydrocephalus characterized by marked ventriculomegaly and domed cranium. Very severely affected hydrocephalic pups exhibited poor weight gain and early death. The control (saline) and Hb-injected pups did not demonstrate hydrocephalus phenotype or significant ventricular enlargement at any postnatal days from Day 5 to Day 26. While ventricular volumes at Day 26 were markedly enlarged in iron- and lysed RBC-injected rat pups compared to saline- and hemoglobin-injected rat pups (*p* < 0.0001), ventriculomegaly was most severe in the iron-injected rat pups. Ependymal and choroid plexus iron deposition (Perls stain) was significantly increased in iron- (*p* < 0.0001) and lysed RBC-injected pups (*p* = 0.024) with hydrocephalus when compared to that in saline-injected pups. SEM revealed denuding of the ependymal cilia and morphologic changes in the choroid plexus of iron- and lysed RBC-injected pups with hydrocephalus.

**Conclusions:**

Intraventricular free iron is critical to the development of PHH following IVH in neonatal rats, while intraventricular Hb did not cause hydrocephalus. PHH is characterized by morphologic changes to the ventricular ependymal cilia and choroid plexus reflecting iron-mediated cellular injury.

**Supplementary Information:**

The online version contains supplementary material available at 10.1186/s12987-025-00745-7.

## Background

Post-hemorrhagic hydrocephalus (PHH) following severe IVH is among the most catastrophic sequelae of preterm birth and a leading contributor to morbidity and mortality in prematurity [1–3]. 40% of infants born prematurely with Grade 4 IVH die during the birth hospitalization [1]. The hemorrhage that occurs in the germinal matrix and within the ventricles during the initial hours to days following preterm birth is known to be the inciting event leading to subsequent hydrocephalus [4–6]. While the mechanisms by which IVH causes subsequent hydrocephalus are incompletely understood, there is evidence for contributions of ependymal ciliary dysfunction, choroid plexus hypersecretion, and glymphatic dysfunction [7–10]. Further, it is not understood why some infants develop post-hemorrhagic ventricular dilation following severe IVH that subsequently resolves and never requires surgery for permanent CSF diversion, while other progress to lifelong shunt-dependent hydrocephalus [11]. It is unclear at what point the pathology diverges between the severe cases that go on to require surgical intervention and those that recover and do not require placement of a shunt for CSF diversion.

**Fig. 1:**
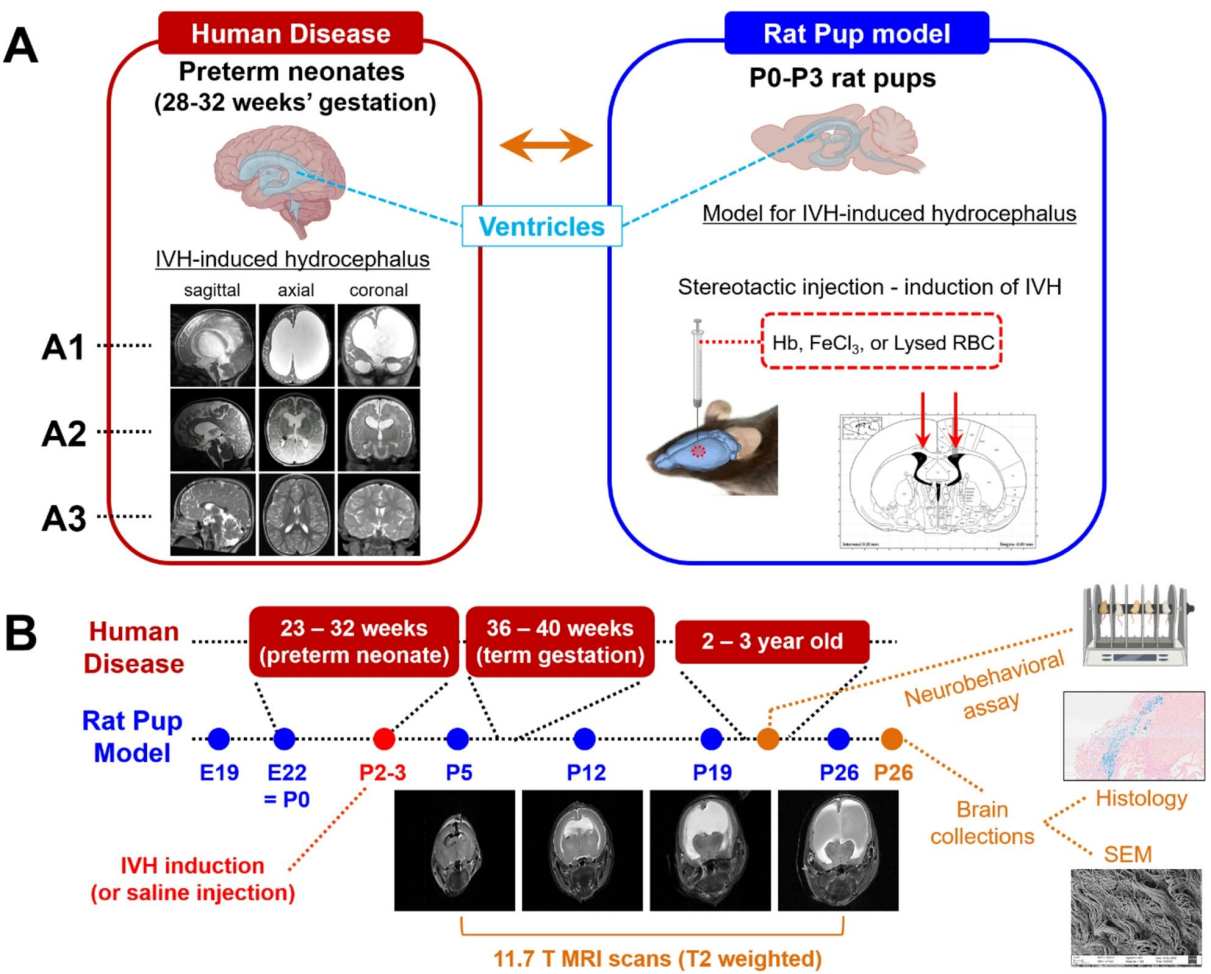
Development of a rat pup IVH-induced hydrocephalus model relevant to human preterm neonatal brain. (**A**) Rat-pup model that recapitulates IVH environment in the brain with a timeframe equivalent to the human preterm neonates (A1) MR scan of a 9-month-old infant with PHH (birth at 26 weeks gestation with a grade IV IVH on day 3 of life) (A2) MR scan of a 4-month-old child with mild-moderate ventriculomegaly consistent with post-hemorrhagic ventricular dilation (PHVD) without hydrocephalus (birth at 23 weeks gestation with a grade IV IVH on day 2 of life) (A3) MR scans of a child without hydrocephalus demonstrating normal ventricular caliber and configuration (**B**) Human gestational ages and the timeline of rat pup experiments: bilateral stereotactic injection for induction of IVH in lateral ventricles at P2-3 followed by MR scans (T2 weighted) of ventricles at P5, P11, P18 and P26, neurobehavioral assays between weeks 2 and 3, and collection of brains at P26 for Perls Stain and SEM

Our prior work has highlighted the association between higher rates of hemoglobin and iron in the cerebrospinal fluid (CSF) following IVH and the subsequent development of hydrocephalus requiring a shunt for CSF diversion [12]. Further, prior studies have shown intraventricular injection of hemoglobin (Hb) or iron to result in ventriculomegaly in rodents [13–16], and we have hypothesized based on our prior work and previous animal studies that iron is the causative agent inducing hydrocephalus following IVH. However, many existing models of neonatal PHH demonstrate a mild to moderate hydrocephalus phenotype which may resolve over time, the human correlate of which may not require neurosurgical intervention. In addition, many existing models neither adequately capture the timing of IVH in the developmentally immature very low birthweight (VLBW) premature infants brain, nor demonstrate a severity of ventricular dilation consistent with severe clinical PHH observed in human infants – which progressed to a lethal condition if left untreated. A few recent animal studies modeled preterm IVH by injecting whole blood or hemolyzed blood into lateral ventricles of rodents between P1-P4 and demonstrated more severe hydrocephalus when compared to previous studies [17, 18]. Those studies support our hypothesis that the timing of IVH is critical to the development of severe PHH. However, it still remains unclear what component of intraventricular blood products following IVH induces severe PHH in premature infants. Thus, there is a need for a preclinical model that simulates severe preterm IVH as closely as possible (injection of blood or blood components) in the early postnatal period, and reproduces both the phenotypic and radiographic features of severe PHH. The long-term goal of our work is to understand the pathogenic mechanisms leading to the development of severe hydrocephalus following preterm IVH and to translate therapies to prevent hydrocephalus following severe preterm IVH. Therefore, we sought to develop a model of preterm IVH resulting in severe PHH – one that would require neurosurgical intervention. Further, we sought to determine the causal element of intraventricular blood degradation product responsible for the development of hydrocephalus follow severe IVH (Fig. 1).

## Methods

All animal experiments were conducted in accordance with protocols approved by the Stanford University Institutional Animal Care and Use Committee (IACUC) and performed in accordance with institutional APLAC guidelines. All surgical procedures were performed with aseptic animal surgical technique.

### Animals

Timed pregnant Sprague Dawley dams were ordered from Charles River laboratories at E16 and were allowed to acclimatize to the animal facility for 1 week prior to giving birth. Two days after birth, the animals were sexed, weighed, and micro-tattooed for identification purposes. Animals were randomized to the experimental group (IVH induction) or control group. Each experimental unit (litter of pups) contained both control animals and experimental (IVH induction) animals from each group, and randomization was completed with equal number of sexes per group to the extent feasible (i.e., accounting for different number of each sex within a litter and variable litter size). Sample size calculations were estimated based on known variability in litter size and anticipated differences in ventricular volumes between groups. On the third postnatal day, intraventricular hemorrhage was induced via bilateral injection (50-80 µL) of either freshly lysed RBCs from their dam (*n* = 41), FeCl_3_ (*n* = 49), or bovine hemoglobin (*n* = 25). Control animals received injection of 7.5 µL/g of sterile saline bilaterally (*n* = 20).

Only animals noted to be deceased or missing prior to the initial survival surgery were excluded from randomization and subsequent experiments. The initial MRI completed at 24 h post-IVH (or saline) injection was evaluated for accuracy of intraventricular injection. Only animals in which the IVH injection did not result in intraventricular injection were excluded from further analysis. Animals were randomized to IVH induction group or control group with equal distribution by sex. The randomization was completed and recorded by the technician completing the animal survival surgeries and randomization was within each litter. Only the technician completing the survival surgery was aware of the randomization group assignment and all subsequent assessments were blinded. The primary outcome measure was defined a priori as ventricular volume differences between groups. The secondary outcomes measures (defined a priori) included neurobehavioral assessments, histological comparison with PERLs stain, and SEM assessments of ultrastructural changes to the ventricular ependyma and choroid plexus.

### Red blood cell (RBC) lysate preparation

The blood lysate was prepared from blood collected from the retro-orbital sinuses in the dams. Briefly, the dams were anesthetized with the isoflurane concentration set at 2% and the oxygen level was set at 1.2 L/min for the induction of anesthesia. Blood was collected using heparinized capillary tubes into DNAse/RNAse free Eppendorf tubes prefilled with sodium citrate (3.8%) buffer at 1:10 volume ratio (buffer: blood). Approximately 1 to 1.1mL of blood was collected and was mixed by turning the tube upside down 3–4 times to prevent coagulation and kept on ice. The blood sample was centrifuged at 10,000 rpm at 4° C for 10 min to remove the plasma. The plasma (supernatant) was clear to light yellow. The red color of plasma indicated the partial lysis of the blood during collection, and we repeated the procedure until we had clear plasma. A recent study demonstrating the FDA-approved pharmacological solvent, DMSO, induced hydrocephalus in a dose-dependent manner [19] highlights the importance of confounding effects on the induction of hydrocephalus. To avoid this concern, we note that the anticoagulant in the blood collection tubing does not remain in the preparation of L-RBCs because the sodium citrate buffer sequestering extracellular/free Ca^2+^ in the blood was dissolved in plasma (0.38%) [20] and removed with plasma during the repeated centrifuging procedures. After the plasma was discarded, an equal volume (to the removed plasma) of DNAse/RNAse free sterile water (UltraPure™ water, Invitrogen 10977) was added for lysis. This was mixed well and then placed in a water bath sonicator for 10–15 min. Then, the sample was briefly vortexed and sonicated again in a water bath sonicator for an additional 5 min. Then, the sample was vortexed again and centrifuged at 2,000 rpm at 4° C for 5 min to separate the blood lysate from membrane fraction. The RBC membrane fraction, which was clumped together after the sonication process, was discarded, leaving behind clear red lysate for the injections. The lysate was carefully transferred into fresh Eppendorf tubes and kept on ice until injected. The hemoglobin concentration in the lysate was measured and recorded using AimStrip^®^Hb hemoglobin meter.

### FeCl_3_ preparation

A 100 mg/mL stock solution was made by dissolving 100 mg of FeCl_3_ (Sigma cat #:157740) in 0.9% saline solution, then sterilized with a 0.22 μm syringe filter. The stock solution was then diluted to 1.2 mg/mL in 0.9% saline solution, which was calculated based on a prior study using a rat model of IVH [13] as well as calculations of the equivalent iron concentrations present in the L-RBC injection group based on volume of injection of 7.5 µL/g, and mean Hb measurements taken from the L-RBC preparations.

### Hemoglobin preparation

120 mg/mL hemoglobin solution was prepared by dissolving 120 mg of bovine hemoglobin (Sigma cat #:H2500) in 0.9% saline solution and sterile filtered with a 0.22 μm syringe filter. The hemoglobin dose selected was based on a prior study using a rat model of IVH to induce hydrocephalus [13].

### Surgical procedure

Rat pups were weighed and briefly anesthetized in the induction chamber with 1.5-3% isoflurane and 1.2 L/min oxygen before being securely placed on the stereotactic frame (Kopf instruments) where anesthesia was maintained at 1.5-3% (depending on resistance of animal to anesthesia). Once secured to the frame the pup was cleaned and prepped using 3 each alternating betadine and alcohol swabs. After confirmation (toe pinch) of adequate anesthesia, a small, < 0.5 cm incision was made along the midline to expose the top of the skull. The incision was then separated with a clean swab and the bregma was located and marked. A prefilled syringe was then secured to the Stoelting microinjection pump. The needle of the syringe was then lined up with the pup’s bregma and zeroed, bilateral injection of the lysate was performed at 0.6 AP, ± 1.00 ML from the bregma at 2.25 DV (AP, ML, and DV; anterior-posterior, medial-lateral, and dorsal-ventral, respectively). The injection volume was determined by the weight of the animal (7.5 µL/gram of animal) with a steady infusion rate of 10 µL/minute, requiring 5–8 min per injection. After injection, the needle was left in place for 4–5 min to reduce backflow. The needle was then slowly removed, and the injection site was observed for injection site leakage, after which the pup was removed from the frame and the wound was closed using suture and/or Vet-Bond surgical adhesive. Pups were then placed in a clean cage with a heating pad to recover and, once fully recovered, they were returned to the dam in pairs to help avoid cannibalism.

### Magnetic Resonance Imaging (MRI)

Ventricular volumes were assessed at postnatal days 5, 12, 19 and 26 with 11.7 T ultra-highfield small animal MRI (Agilent Technologies, Inc. CA) in the Canary Center at the Stanford School of Medicine. Rat pups were anesthetized with isoflurane and placed on the bed in the radiofrequency (RF) volume coil of the MR machine during the scanning of brains. The mouse coil was used for scans of week 1 and 2 rat pups, and the rat coil was used for scans of week 3 and 4 rat pups. T2-weighted images in the coronal plane were obtained for all animals to evaluate the area of CSF in the ventricles, which was extracted by MicroDicom (MicroDicom Ltd.) and quantified by ImageJ (NIH). The ventricular CSF volumes at D5, D12, D19, and D26 were quantified by multiplying the area of the ventricle by the MRI slice thickness. The number of animals used for calculating ventricular CSF volumes were 20, 25, 49, and 41 for the control, Hb, iron, and L-RBC injection group, respectively. Two animals from the L-RBC group at D19 were excluded from the analysis because the MRI machine was unavailable (technical malfunction) when scanning the pups on D19 (*n* = 39). These animals were included for analysis on D26 when the MRI machine was back in function (*n* = 41). For ventricular volume measurements, we included CSF volumes taken from measurements of the lateral and 3rd ventricles, which were consistently captured in all MR images. As the cerebral aqueduct and 4th ventricles were not consistently imaged in every scan, we excluded these CSF spaces as well as the extra-axial subarachnoid spaces from our ventricular volume measurements.

### Neurobehavioral assessments

Neurobehavioral assessments were completed at postnatal day 21. Hindlimb support test, ascending rope test, and rotarod testing were completed. In addition, clinical and behavioral manifestations of hydrocephalus, including poor weight gain, lethargy, and failure to thrive were monitored.

### Hindlimb support test

A 2-mm thick and 70-cm long wire was extended horizontally between two poles at 50 cm height. The experimenter held the animal by the nape of its neck and let its forepaws touch the wire. Grasping usually ensued immediately, followed by tensing of the shoulder muscles, attempts at pull-ups with the forelimbs, and the synergistic support of the body with the hindlimbs. Animals were scored by use of one or both limbs to help support the mselves [21].

### Ascending rope test

A 17-mm wide wooden rod and a rope of the same width were positioned in the center of a container filled with cold water (15 °C). Connected with the rod and rope was a platform 20 cm above the water level. If an animal fell into the water, it was left in it for 30s; it was then aided up to the platform with the experimenter’s assistance. Falls, climbing time, and successful landing (within 180 s) were recorded. Record of falls, climbs, and successful landing were made [22].

### Rotarod test

Animals were assessed on the Rotarod over 3 trials with a 15-minute rest period between trials at P21. Each trial started with an accelerating rotation (10 rpm for 15 s, 20 rpm for 15 s, and 30 rpm until the animal fell or 180 s passed). The time was recorded when the animal fell or the maximum trial time was finished (180 s) [23].

### Scanning electron microscopy (SEM) of ependyma and choroid plexus

Brain samples were collected immediately after the last MR scans at D26 (23D post-injection) from experimental and control animals in ice cold 1X PBS. Brains were then carefully dissected to open the lateral ventricle and a section of ventricular ependyma and choroid plexus was removed and fixed (0.1 M sodium cacodylate, 2% glutaraldehyde, 4% paraformaldehyde [PFA]) overnight. The fixative was then removed, and samples were washed in 0.1 M sodium cacodylate and then stained with cold 1% osmium tetroxide for 1 h. The samples were then dehydrated via increasing ethanol concentration washes (50–100%). The samples were chemically dried using hexamethyldisilazane. Once dried, the brain samples were mounted on a carbon-backed stub and sputter coated with 4–5 nm of gold using the Leica ACE600. They were then imaged at the Stanford University Cell Sciences Imaging Core Facility (RRID: SCR_017787) Zeiss Sigma FESEM.

### Perls Prussian blue stain (iron stain) and image analysis

Brain samples were collected immediately after the last MR scans at D26 (23D post-injection) in 4% PFA overnight and were sectioned coronally with a 1-cm thickness at the region of the lateral ventricles. Sectioned tissue was sent to the Histo-Tec Laboratory (https://www.histoteclab.com/) for iron stain (Perls Prussian blue stain). The stained samples on microscope slides were scanned with Aperio AT2 scanner (Leica), and the view showing the whole brain region was captured from each sample using the Aperio ImageScope software (Leica, version 12.4.3). The captured images were used for the “blue/pink analysis” that we developed with the LabView programming language to determine the ratio of the iron deposition area (blue) over the whole brain tissue area. The procedures for the “blue/pink analysis” are illustrated in Fig. S1. Briefly, the identification of the blue (iron stain)/pink (eosin stain) area is a set of three thresholds in 3D space of RGB color code from the histology images. The blue/pink ratio was determined empirically by adjustment of threshold for three color (RGB) code sets and was applied to each image. The total number of “blue” pixels was divided by the total number of “blue” and “pink” pixels for quantitative comparison of iron deposition among groups with saline, Hb, iron, and L-RBC injections. Our LabView stand-alone executable application is available upon request for use only in non-profit research, with acknowledgement of the current paper.

### Statistical analysis

All statistical tests were conducted using Prism (GraphPad) software unless otherwise specified. Gaussian distribution was examined by the Shapiro–Wilk normality test. For parametric data (normality test passed), unpaired two-tailed Student’s t-tests or one-way ANOVA tests with Tukey’s post hoc correction were performed to determine the statistical significance (*p* < 0.05). When standard deviations were not equal (*p*_F−test_ < 0.05), Brown-Forsythe and Welch ANOVA tests with Dunnett’s post hoc correction were performed. For non-parametric data (normality test not passed), two-sided unpaired Mann–Whitney tests or Kruskal-Wallis tests with Dunn’s post hoc correction were used to determine the statistical significance (*p* < 0.05). For comparison of changes in CSF volumes among groups from D5 through D26, the linear regression analysis was performed to determine the Pearson’s correlation coefficient (r) and the slope. For all animal experiments, the number of independent rat pups utilized is noted in figure legends and the Results section.

## Results

### Iron (Fe^3+^) and lysed RBC deformed the rat pup cranium and increased ventricular volumes

**Fig. 2:**
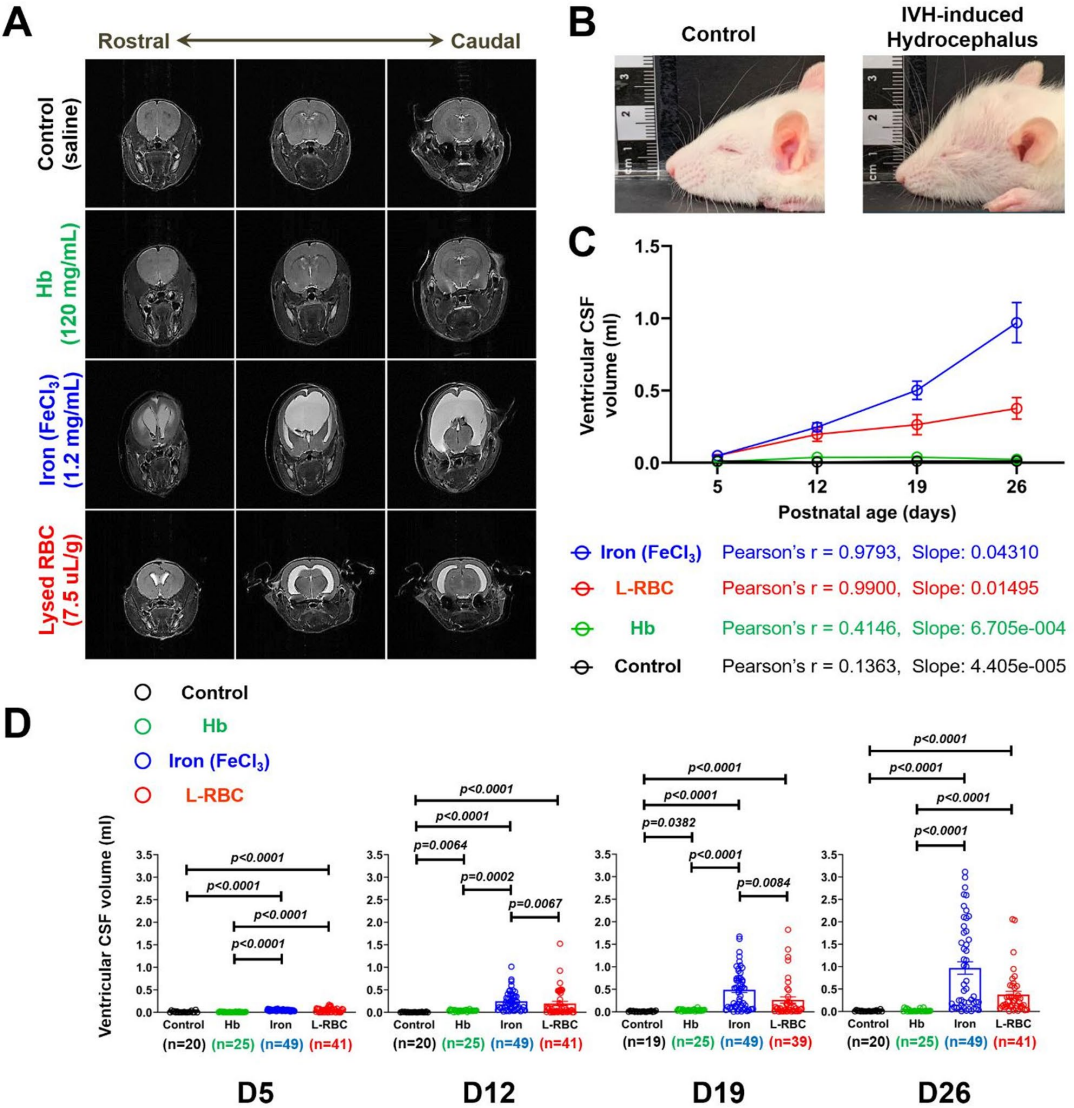
Ventricular enlargement and resultant domed cranium in IVH-induced hydrocephalus. (**A**) Comparison of T2-weighted MRI demonstrating ventricular CSF volumes at 3 weeks (D26) after stereotactic injection of blood breakdown products at postnatal day 3. Ferric iron (Fe^3+^) and L-RBC injection resulted in global ventriculomegaly – visualized from rostral to caudal. (**B**) Rat pups with severe IVH-induced hydrocephalus developed a domed cranium. (**C**) Between-groups comparison of time-trends of ventricular CSF volumes from D5 to D26: Linear regression analysis showed the changes in CSF volumes of the iron and L-RBC injection groups had strong linear relationship (*r* = 0.9793 and 0.9900, respectively) and the slopes of the iron (0.04310) and L-RBC (0.01495) groups were significantly different from the control (0.00004405) and Hb (0.0006705) injection groups (*p* < 0.00001). (**D**) Ventricular CSF volumes were assessed from the MR images. Ventricular CSF volumes of iron-and L-RBC-injected rat pups at all time points (D5 to D26) were significantly increased compared to saline- (control) and Hb-injected rat pups (*p* < 0.0001, Kruskal-Wallis tests with Dunn’s post-hoc correction). The control and Hb groups showed distinct CSF volumes from D12 to D19 (*p* = 0.0064 at D12, *p* = 0.0382 at D19), but then there was no significant difference at D26 (*p* > 0.9999). The mean values of ventricular CSF volumes from D5 → D12 → D19 → D26: control (0.01208 → 0.005668 → 0.0105 → 0.0115 mL), Hb (0.007613 → 0.03797 → 0.03839 → 0.02312 mL), iron (0.04906 → 0.2462 → 0.5019 → 0.9695 mL), L-RBC (0.04934 → 0.1970 → 0.2635 → 0.3760 mL), respectively

Rat pups injected with iron and L-RBC developed marked progressive ventriculomegaly over the course of 3 weeks - demonstrated on serial MR imaging (11.7 T) (Fig. 2A). The ventricles including the lateral and 3rd were most consistently and severely enlarged in the iron injection rat pups (0.9695 ± 0.1386 mL, mean ± SEM) at D26. Control (saline injection) (0.0115 ± 0.0030 mL, mean ± SEM) and Hb injection (0.02312 ± 0.0057 mL, mean ± SEM) rat pups did not demonstrate significant ventricular enlargement until D26. Rat pups with severe hydrocephalus developed a domed cranium. This deformation of the skull is caused by the increase of CSF volumes within the brain exerting outward pressure on the growing skull (Fig. 2B). MRI ventricular volume measurements were taken from rostral to caudal including the lateral and 3rd ventricles in each animal from week 1 to 3. The final MRI was obtained prior to sacrifice at D26. In cases of death from hydrocephalus, the final MRI was prior to D26. To determine the progressive changes in the ventricular CSF volumes following IVH induction, we traced the ventricular CSF volumes at all time points (D5, D12, D19, and D26) for the control (*n* = 20), Hb (*n* = 25), iron (*n* = 49), and L-RBC (*n* = 41 for D5, D12, and D26, *n* = 39 for D19) groups. Both the iron and L-RBC injection groups showed progressive increase in the ventricular CSF volumes (slope = 0.04310 and 0.01495, respectively) with strong linearity (Pearson’s *r* = 0.9793 and 0.9900, respectively) whereas the control and Hb injection groups did not show progressive changes in the ventricular CSF volumes (slope = 0.00004405 and 0.0006705, respectively) (Fig. 2C). The ventricular CSF volumes were significantly increased in rat pups with IVH induced by iron (D5, D12, D19, and D26: 0.04906, 0.2462, 0.5019, and 0.9695 mL, respectively) or L-RBC injection (D5, D12, D19, and D26: 0.04934, 0.1970, 0.2635, and 0.3760 mL, respectively) compared to saline (D5, D12, D19, and D26: 0.01208, 0.005668, 0.0105, and 0.0115 mL, respectively) and Hb (D5, D12, D19, and D26: 0.007613, 0.03797, 0.03839, and 0.02312 mL, respectively) rat pups at all time points from D5 through D26 (p _(vs. Control)_ < 0.0001, p _(vs. Hb)_ < 0.0001) (Fig. 2D). Hb injection (0.03797 mL at D12, 0.03839 mL at D19) resulted in increased CSF ventricular volumes compared to saline injection (0.01208 mL at D12, 0.005668 mL at D19) between D12 and D19 (p _(vs. Control)_ = 0.0064 at D12, p _(vs. Control)_ = 0.0382 at D19), but at D26 there was no significant difference in the CSF volume between the control and Hb injections (p _(Control vs. Hb)_ > 0.9999).

### Iron (Fe^3+^) and lysed RBC induced denudation of ependymal cilia and altered surface morphology of choroid plexus epithelium

**Fig. 3:**
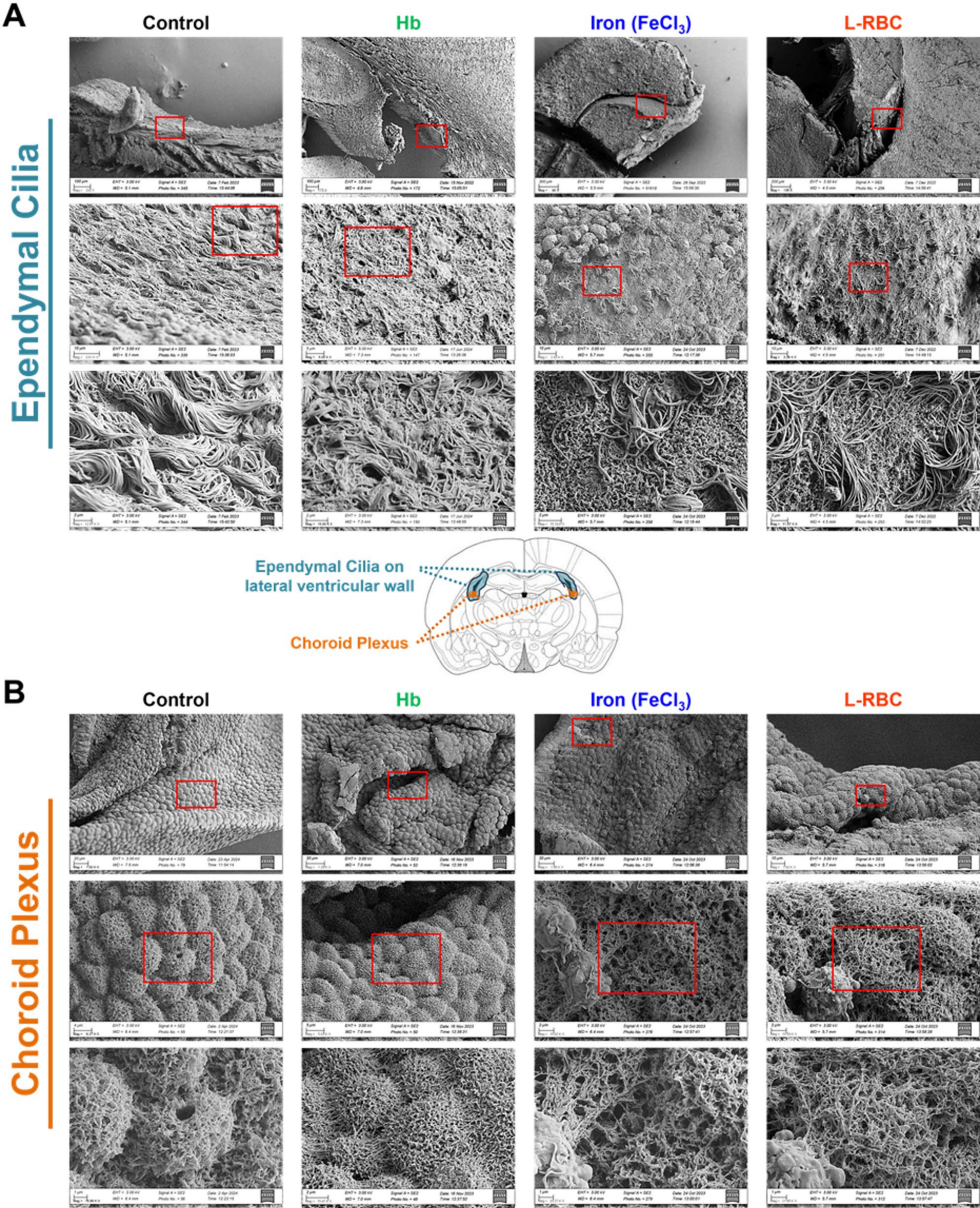
SEM images showing the altered surface morphology of lateral ventricles in post-hemorrhagic hydrocephalus. (**A**) Ependymal cilia on the lateral ventricular wall at D26 (23D post-injection). Iron and L-RBC injections caused significant loss of ependymal cilia (i.e., denudation) when compared to the saline and Hb injections as shown in the second and third row images. (**B**) The surface of the choroid plexus at D26 (23D post-injection): Iron and L-RBC injections caused the surface of cells to be more porous and reticulated when compared to saline injections

We examined the CSF-facing tissues – the ventricular ependyma and choroid plexus epithelium with SEM to evaluate the effect of post-hemorrhagic hydrocephalus. SEM images revealed that iron and L-RBC injections caused significant loss of ependymal cilia (i.e., denudation) on the surface of lateral ventricular wall when compared to the saline and Hb injection groups as shown in the SEM images on the second and third rows of Fig. 3A. Iron and L-RBC injections also altered the surface morphology of choroid plexus (Fig. 3B). The surface of the choroid plexus in the iron- and L-RBC- injected rat pups demonstrated more porous and reticulated structures compared to the choroid plexus of saline- and Hb-injected rat pups.

### Iron injections significantly increased ependymal and choroid plexus ferric iron (Fe^3+^) deposition

**Fig. 4:**
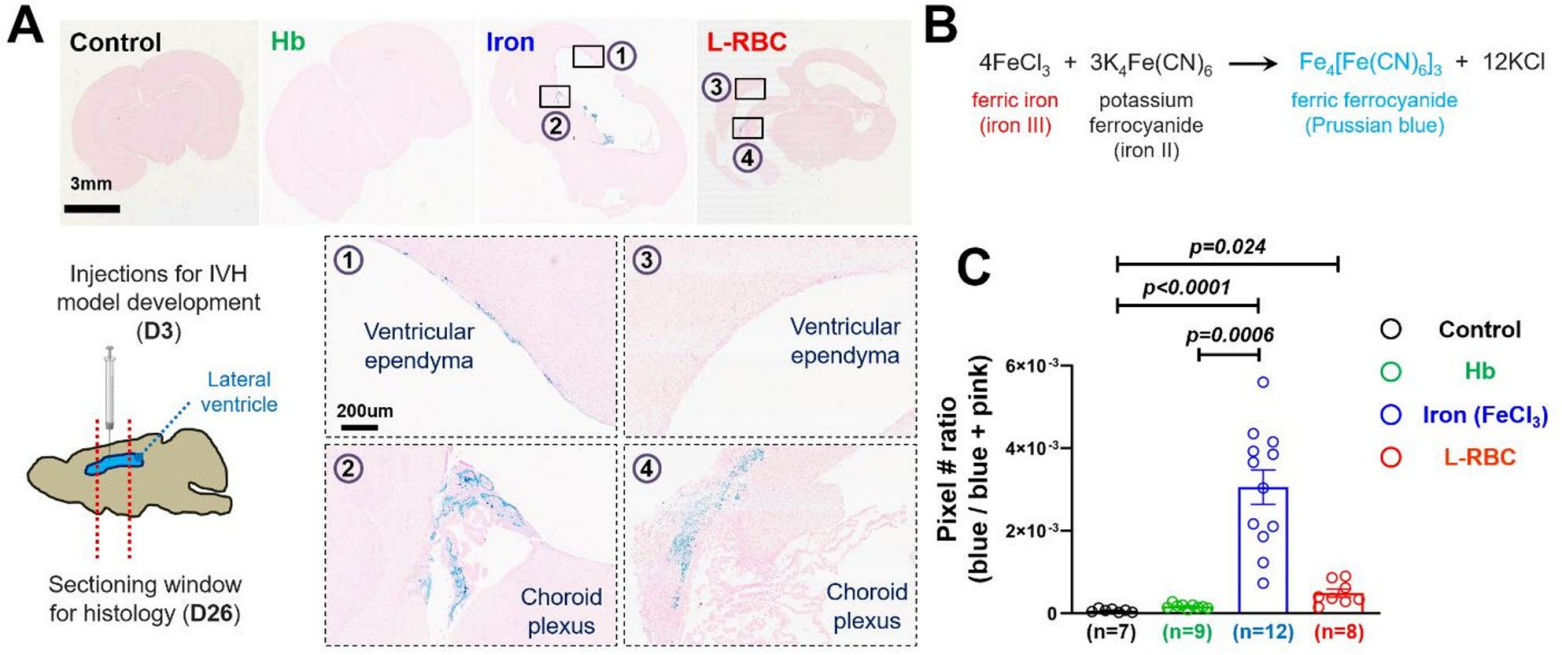
Iron deposition in the lateral ventricles following IVH induction. (**A**) Perls Prussian blue stain images showing iron deposition (blue) on the wall of lateral ventricles (ependyma) and choroid plexus three weeks after injections of saline (control), Hb, Iron, and L-RBC (D26: 23 days post-injection). The sagittal brain illustration demonstrates the sectioning window of the lateral ventricle collected for histology analysis. Iron injections involved strong blue stain in both (1) ventricular ependyma and (2) choroid plexus whereas L-RBC injections involved strong blue stain only around (4) choroid plexus, but not in (3) ependyma (**B**) Ferric iron (Fe^3+^) forms insoluble Prussian blue pigment (i.e., ferric ferrocyanide) by reacting with soluble ferrocyanide (**C**) Quantification of iron deposition levels. Image analysis using the LabView code measured the ratio of the “blue” stain area to the total tissue area. Iron injections significantly increased the deposition of ferric iron when compared to saline (*p* < 0.0001) and Hb (*p* = 0.0006) injections. L-RBC injections significantly increased the ferric iron deposition when compared to saline (*p* = 0.024). Iron and L-RBC injections did not differ in iron deposition (*p* = 0.2026), nor did Hb and L-RBC injections (*p* = 0.7360). Statistical significance was set at *p* = 0.05. (Mean values of blue/pink pixel ratios (unitless): control (0.661 × 10^− 4^), Hb (1.679 × 10^− 4^), iron (30.54 × 10^− 4^), L-RBC (4.882 × 10^− 4^), Kruskal-Wallis tests with Dunn’s post-hoc correction)

To investigate if ferric iron deposition within the ventricles is evident in post-hemorrhagic hydrocephalus, we performed Perls Prussian blue stain on the histologic slices of rat pup brains injected with blood breakdown products. Both iron and L-RBC injections demonstrated strong Perls stain in the choroid plexus whereas saline and Hb injections rarely exhibited appreciable blue stain in any location of lateral ventricle and choroid plexus **(**Fig. 4A**)**. The iron injection group also demonstrated strong Perls stain in ventricular ependyma while the L-RBC injection groups showed weak blue stain in ventricular ependyma. The area staining avidly with blue demonstrates ferric iron (Fe^3+^) deposition from the reaction of ferric iron with ferrocyanide, forming the Prussian blue pigment (Fig. 4B). Quantification using our LabView code revealed that the iron injection (30.54 ± 4.17 × 10^− 4^, unitless mean ± SEM) significantly increased the area of ferric iron deposition in the ventricular ependyma and choroid plexus compared to saline (0.661 ± 0.147 × 10^− 4^, unitless mean ± SEM) (p _(control vs. Iron)_ < 0.0001) and Hb injections (1.679 ± 0.183 × 10^− 4^, unitless mean ± SEM) (p _(Hb vs. Iron)_ = 0.0006) (Fig. 4C). L-RBC injection (4.882 ± 0.974 × 10^− 4^, unitless mean ± SEM) increased the area of iron deposition when compared to the saline injection group (p _(control vs. L−RBC)_ = 0.024), but did not show statistical significance when compared to the Hb injection group (p _(Hb vs. L−RBC)_ = 0.736). Iron deposition did not differ significantly between the iron-injection and L-RBC-injection rat pups (p _(Iron vs. L−RBC)_ = 0.2026).

### Neurobehavioral assessments did not correlate with IVH induction group or hydrocephalus status

**Fig. 5:**
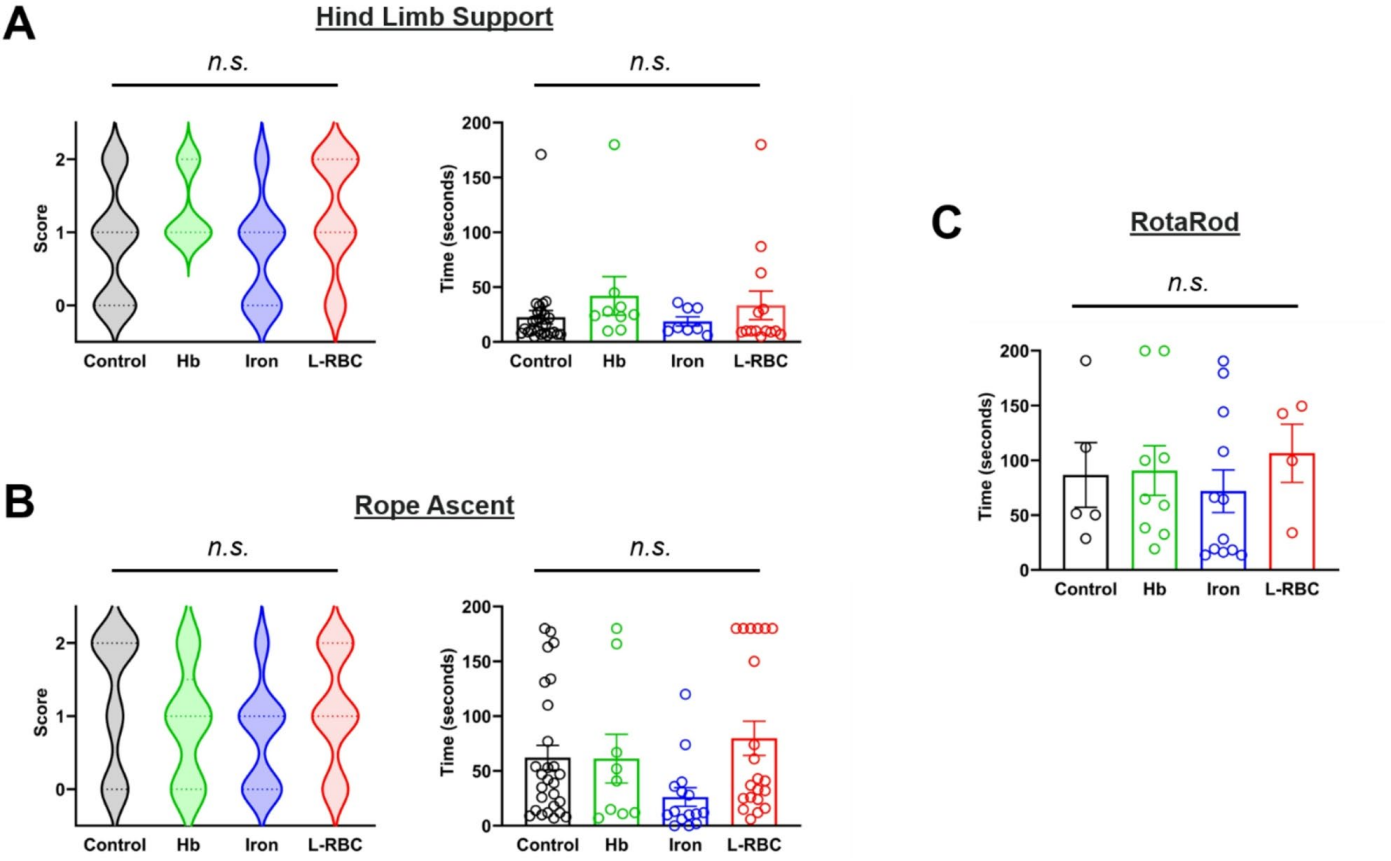
Neurobehavioral assessments in correlation with IVH induction group or hydrocephalus status. We performed neurobehavioral assessments to test if the PHH-developing rat pups correlated with neurobehavioral deficits. A battery of neurobehavioral assessments including a hind limb support test, rope ascent test, and rotarod test was analyzed in terms of IVH induction group. (**A**) Hind limb support test showed that there was no statistical significance in the score and the time of grasping the wire with hindlimbs among saline (*n* = 27), Hb (*n* = 9), iron (*n* = 15), and L-RBC (*n* = 21) groups. (**B**) Rope ascent test showed that there was no statistical significance in the score and the time of climbing and landing on the platform among the four groups. (**C**) Rotarod test showed that there was no statistical significance in the time of fall-off on a rotating rod among saline (*n* = 5), Hb (*n* = 9), iron (*n* = 12), and L-RBC (*n* = 4) groups. (Statistical significance: *p* < 0.05 with Kruskal-Wallis tests with Dunn’s post-hoc correction)

We performed a battery of neurobehavioral assessments to determine if PHH rat pups develop neurobehavioral deficits. We performed hind limb support test [22], rope ascent test [21], and rotarod test [23] (Fig. 5), which had been used to evaluate abnormal neurobehavior in rat IVH models. We analyzed the data according to the treatment group (saline, Hb, iron, and L-RBC) (Fig. 5). No statistically significant between-group differences in neurobehavioral assessments were found for any of the tests. For the hind limb support test, the groups did not show significant difference in either the score or the duration time of grasping the wire with one or two hindlimbs (Fig. 5A). For the rope ascent test, the groups did not show significant difference in either the score of climbing the rope or the time to successful landing on the top of the platform (Fig. 5B). For the rotarod test, there was no statistical significance among the groups in time to falling from a rotating rod (Fig. 5C). While we failed to distinguish PHH with neurobehavioral deficits, we did note that pups with severe PHH exhibited lethargy and slowed movements when compared to pups with mild ventriculomegaly or normal CSF volumes. In addition, many pups that began to exhibit lethargy then died in the subsequent days, prior to the end of the 25-day experiment (Video S1).

## Discussion

There is an urgent need to develop therapies to prevent hydrocephalus following severe IVH. The current treatment paradigm relies on invasive CSF diversion surgery once the disease is already advanced [1, 2, 5, 24]. A significant hurdle to translating new therapies is the ability to test disease mechanisms and novel therapeutics in a model that recapitulates the severe hydrocephalus phenotype observed in infants, which necessitates neurosurgical intervention. While existing models of mild ventriculomegaly following IVH likely have relevance to the underlying disease state, there is clearly a difference between a mild-moderate ventriculomegaly phenotype that may never progress to require neurosurgical intervention and a severe hydrocephalus phenotype that requires surgical intervention for permanent CSF diversion. To this end, we developed an animal model of severe hydrocephalus by testing IVH induction by different blood breakdown products. We induced IVH at the time-frame equivalent to human preterm neonates (< 33 weeks), reflecting the age at which preterm neonates are at highest risk for IVH from the germinal matrix [6, 24–28]. Our model produced a severe and progressive ventriculomegaly with clinical phenotype mirroring human clinical disease progression (i.e., progressive macrocephaly, lethargy, risk of death when left untreated). Our model also clearly demonstrated that intraventricular iron causes severe and progressive hydrocephalus during the whole period of our monitoring with MR scans when compared to the control and Hb groups with high statistical significance (*p* < 0.0001). The findings were similar, though less severe, in the lysed red blood cells (L-RBC) group. These findings are consistent with our prior work demonstrating higher levels of CSF iron and ferritin in infants with severe early ventricular enlargement following IVH, compared to those with moderate early ventricular enlargement [29].

However, our findings contrast with published findings indicating that intraventricular Hb causes hydrocephalus at postnatal day 4 [13, 30, 31]. There are several possible explanations for this discrepancy. Gestational age at time of IVH induction may play a role, as it has been shown that IVH induction at earlier postnatal age (postnatal day 2 compared to postnatal day 5) in rats is associated with a protective effect against innate immune activation, and alterations in white matter and cerebral ventriculomegaly [32]. In addition, the degree of ventriculomegaly observed in hydrocephalus induced with Hb at postnatal day 4 was less severe than the iron-induced hydrocephalus we observed at postnatal day 3. It is plausible that our model executed at postnatal day 4 would have demonstrated a similar moderate ventriculomegaly in Hb rat pups. Taken together, these findings suggest that different components of blood degradation products may play different roles in the pathogenesis of post-hemorrhagic hydrocephalus. Hydrocephalus induced by Hb injection may depend on innate immune activation, while the effects of intraventricular iron may be independent of immune response. Ferroptosis, an iron-dependent form of non-apoptotic cell death, may play a key role in the pathogenesis of hydrocephalus following IVH. Previous animal work has highlighted the potential of deferoxamine to attenuate ventriculomegaly induced by iron or Hb injection [13, 14]. It is plausible that this effect is due to iron chelation as well as prevention of immune cell activation by deferoxamine and further supports a role for iron-mediated injury contributing to hydrocephalus.

This study not only demonstrates that intraventricular iron is pathogenic to hydrocephalus development but demonstrates that alteration of ventricular ependyma and choroid plexus morphology characterizes this process. It is known that ciliopathies that affect the morphology of the ependymal cilia result in hydrocephalus [8, 10]. The ependymal cilia are thought to play a role in bulk flow of CSF. Alterations in this physiology likely lead to buildup of CSF and outward pressure of accumulating CSF in the ventricular cavities. Morphologic changes to the choroid plexus following IVH has been less extensively studied. However, it has been shown that ion transporters in the choroid plexus are altered following neonatal IVH, resulting in CSF hypersecretion [9, 33, 34]. It is not surprising that choroid plexus morphology is altered in post-hemorrhagic hydrocephalus. This work demonstrates clearly that the morphologic change is a direct result of intraventricular iron. Ferroptosis may play a role in the cell death occurring after IVH resulting in the morphological changes we observed in both the ventricular ependyma and choroid plexus epithelium.

The findings of our MRI, SEM, and Perls stain analyses demonstrate that intraventricular free ferric iron is critical to the development of PHH following IVH. We hypothesize that ferroptosis, an iron-dependent regulated cell death pathway [35, 36], is the mechanism underlying the pathogenic effect of free iron and may be a critical contributor to the development of hydrocephalus following preterm IVH. This is supported by our prior findings of elevated CSF ferritin following high grade IVH [12, 29]. Further evidence in support of this hypothesis is the finding of a significant increase and decrease in two ferroptosis-specific genes, ACSL4 and SLC7A11, respectively in the presence of post-hemorrhagic hydrocephalus [37]. Additionally, removal of iron from ferritin but not hemoglobin has been shown to attenuate hydrocephalus following IVH [38]. Further investigation into this hypothesis is necessary to delineate the mechanism driving iron-mediated PHH.

Interestingly, our neurobehavioral assessments demonstrated no significant difference in hind limb support, rope ascent, and rotarod among saline, Hb, iron and L-RBC groups. This contrasts with the severe morphologic, imaging, and clinical characteristics of hydrocephalus (i.e., MRI, SEM, Perls stain, cranial doming) we observed in rat pups following iron and L-RBC injections. This may be attributed to the inadequacy of current neurobehavioral assessment paradigms in capturing the clinical phenotype of hydrocephalus. Other studies have observed that identifying the origins of behavioral abnormalities is challenging, and thus results from neurobehavioral assessments are not always consistent with pathological severity of hydrocephalus in animal models [22, 39, 40]. While we did observe animals demonstrating lethargy, decreased activity, and focal motor deficits, on an individual basis (**Movie** S1), this was observed more frequently on a case-by-case basis and was not captured systematically by our more structured neurobehavioral assessments. In addition, usually these altered behaviors were noted later in the disease state and in some cases when the hydrocephalus was severe enough that a rat pup was approaching death from hydrocephalus (or meeting euthanasia criteria). Alternatively, delayed neurobehavior assessments (at later time frame/age) may reveal differences we did not observe in this study [22].

Taken together, we developed a rat pup model of post-IVH hydrocephalus that reflects the developmental vulnerability of the preterm neonatal brain and demonstrates a severe hydrocephalus phenotype. This in vivo experimental model will facilitate studying the mechanism underlying PHH. Further, we have identified intraventricular iron as pathogenic to preterm post-IVH hydrocephalus. Future studies will focus on delineating the mechanism by which intraventricular iron causes hydrocephalus as well as test therapeutic strategies (iron chelation, ferroptosis inhibition) to prevent hydrocephalus following severe IVH.

### Limitations

There is clearly much work to be done to understand *how* iron leads to development of hydrocephalus following IVH. Further, recent evidence has made it clear that hydrocephalus following IVH is not simply a problem with CSF absorption, but that CSF hypersecretion is a key feature [9, 41, 42]. How iron-mediated pathology is related to altered ion transport following IVH is yet unclear and is an area for future investigation. In addition, we observed within-group variability in the degree of progressive ventriculomegaly monitored over time in response to iron or L-RBC injection, which is an area for further investigation. As this work is preliminary, we recognize that ciliary disruption is not the only contributor to the development of PHH and that there are other drivers underlying PHH pathogenesis that are appropriate targets for future studies [42, 43]. McAllister et al. have demonstrated how the ventricular and sub-ventricular zones are damaged in PHH and involve significant cognitive implications [44], further supporting the in vitro studies by Castaneyra-Ruiz et al. [45].

## Conclusions

Intraventricular free iron is critical to the development of PHH following IVH, while intraventricular Hb did not cause hydrocephalus. PHH is characterized by morphologic changes to the ventricular ependymal cilia and choroid plexus reflecting iron-mediated cellular injury. Further work is necessary to delineate the cellular mechanisms of iron-mediated injury resulting in hydrocephalus.

## Supplementary Information

Below is the link to the electronic supplementary material.

Supplementary Material 1

Supplementary Material 2

## Data Availability

The data generated and/or analyzed during the current study are available from the corresponding author on reasonable request.

## Code availability

The LabVIEW code for the analysis of histology images is available at https://github.com/HadrianBT/Hydrocephalus.
