# Supplementary material for: Intraventricular iron causes severe hydrocephalus – a model of severe neonatal hydrocephalus
Source: Fluids Barriers CNS. 2025 Dec 24;23:16. doi: 10.1186/s12987-025-00745-7 (PMC12837436; doi:10.1186/s12987-025-00745-7)
Supplement: Supplementary file 1 — Supplementary Material 1 [file 12987_2025_745_MOESM1_ESM.docx]

**(Supplementary Materials)**

**Intraventricular iron causes severe hydrocephalus – a model of severe neonatal hydrocephalus**

**Authors:** Kwang-Min Kim, PhD^1,2^, Arokoruba Oboba Cheetham-West, BS^1^, Mohamed Rafiuddin Ahmed^3^, PhD, Megan Phillips, BS^1^, Andrey V. Malkovskiy, PhD^4^, Venkata Raveendra Pothineni, PhD^5^, Kyle Brewer, PhD^5,9^, Chirag B. Patel, MD, PhD^6,7,8^, Jayakumar Rajadas, PhD^5,9^, Kelly B. Mahaney, MD, MS^1,*^

**Affiliations:**

^1^ Stanford University School of Medicine, Department of Neurosurgery; Stanford, CA 94305, USA

^2^ Gachon University College of Medicine, Department of Physiology; Incheon 21999, Korea

^3^ Vanderbilt University, Department of Pharmacology; Nashville, TN 37232, USA

^4^ Carnegie Institute of Washington, Department of Plant Biology; Stanford, CA 94305, USA

^5^ Stanford University School of Medicine, Department of Medicine; Stanford, CA 94305, USA

^6^ The University of Texas MD Anderson Cancer Center, Department of Neuro-Oncology; Houston, Texas, 77030, USA

^7^ The University of Texas MD Anderson Cancer Center/The University of Texas Health Science Center at Houston Graduate School of Biomedical Sciences, Cancer Biology Program; Houston, Texas, 77030, USA

^8^ The University of Texas MD Anderson Cancer Center/The University of Texas Health Science Center at Houston Graduate School of Biomedical Sciences, Neuroscience Graduate Program; Houston, Texas, 77030, USA

^9^ Advanced Drug Delivery and Regenerative Biomaterials Laboratory, Stanford University School of Medicine, Cardiovascular Institute; Stanford, CA 94305, USA

*Corresponding author. Kelly B Mahaney, MD, MS. kmahaney@stanford.edu


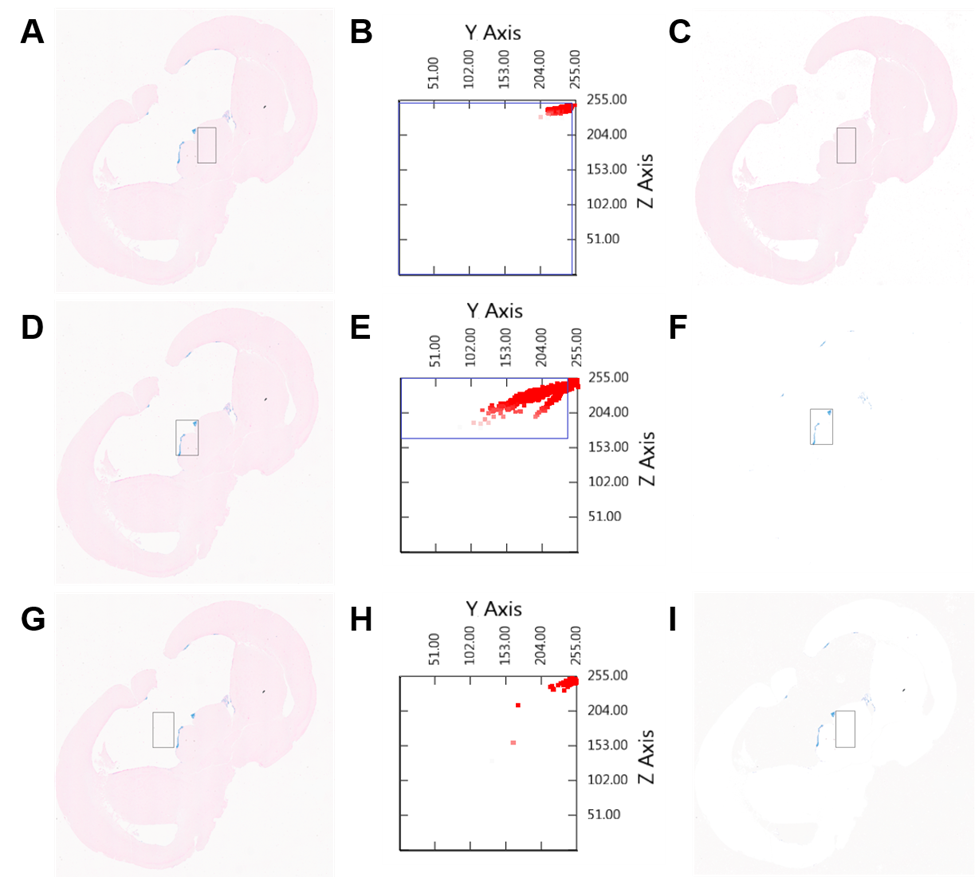


**Fig. S1. Analysis of the blue/pink stain area to determine the level of iron deposition using the LabView Code.**  The blue (iron stain)/pink (eosin stain) area was identified by a set of three thresholds in RGB color code from the Perls Stain images. The values for threshold were determined empirically (A, B) To select pink areas, we kept pixels above, below, and below the thresholds for “R”, “G”, and “B”, respectively. The Y axis indicates “G” and the Z axis indicates “B” in the plot. (C) The resulting image for the pink stain after applying thresholds (D, E) To select blue areas, we kept pixels below, below, and above the thresholds for “R”, “G”, and “B”, respectively. (F) The resulting image for the blue stain after applying thresholds (G, H, I) The background pixels are usually a shade of gray and have very high values in “G” and “B”. Thus, we kept pixels below, above, and above the thresholds for “R”, “G”, and “B”, respectively. This shows pixels that correspond to the inverse of (C).
